# Grouper TRAF4, a Novel, CP-Interacting Protein That Promotes Red-Spotted Grouper Nervous Necrosis Virus Replication

**DOI:** 10.3390/ijms22116136

**Published:** 2021-06-07

**Authors:** Siting Wu, Mengshi Sun, Xin Zhang, Jiaming Liao, Mengke Liu, Qiwei Qin, Jingguang Wei

**Affiliations:** 1 Guangdong Laboratory for Lingnan Modern Agriculture, Joint Laboratory of Guangdong Province and Hong Kong Region on Marine Bioresource Conservation and Exploitation, College of Marine Sciences, South China Agricultural University, Guangzhou 510642, China; sting23332021@163.com (S.W.); 13803898210@163.com (M.S.); 18841525211@163.com (X.Z.); 13360863582@163.com (J.L.); lmkqjh0618@163.com (M.L.); 2 Laboratory for Marine Biology and Biotechnology, Qingdao National Laboratory for Marine Science and Technology, Qingdao 266000, China

**Keywords:** *Epinephelus coioides*, TRAF4, RGNNV, cellular localization, viral replication

## Abstract

Tumor necrosis factor receptor-associated factors (TRAFs) play important roles in the biological processes of immune regulation, the inflammatory response, and apoptosis. TRAF4 belongs to the TRAF family and plays a major role in many biological processes. Compared with other TRAF proteins, the functions of TRAF4 in teleosts have been largely unknown. In the present study, the TRAF4 homologue (EcTRAF4) of the orange-spotted grouper was characterized. EcTRAF4 consisted of 1413 bp encoding a 471-amino-acid protein, and the predicted molecular mass was 54.27 kDa. EcTRAF4 shares 99.79% of its identity with TRAF4 of the giant grouper (*E. lanceolatus*). EcTRAF4 transcripts were ubiquitously and differentially expressed in all the examined tissues. EcTRAF4 expression in GS cells was significantly upregulated after stimulation with red-spotted grouper nervous necrosis virus (RGNNV). EcTRAF4 protein was distributed in the cytoplasm of GS cells. Overexpressed EcTRAF4 promoted RGNNV replication during viral infection in vitro. Yeast two-hybrid and coimmunoprecipitation assays showed that EcTRAF4 interacted with the coat protein (CP) of RGNNV. EcTRAF4 inhibited the activation of IFN3, IFN-stimulated response element (ISRE), and nuclear factor-κB (NF-κB). Overexpressed EcTRAF4 also reduced the expression of interferon (IFN)-related molecules and pro-inflammatory factors. Together, these results demonstrate that EcTRAF4 plays crucial roles in RGNNV infection.

## 1. Introduction

Grouper, *Epinephelus* spp., is one of the most important marine aquaculture fish species in China [1]. The orange-spotted grouper, *Epinephelus coioides*, is a popular marine fish cultured in Southeast Asia and China. However, for many years, outbreaks of infectious bacterial and viral diseases have seriously affected the grouper aquaculture industry, causing large economic losses [2,3]. Larval and juvenile grouper are susceptible to fatal epidemic outbreaks of diseases caused by infections with *Betanodavirus* or nervous necrosis viruses (NNVs) and iridoviruses [2,3,4]. NNVs are some of the most destructive viruses of cultured marine fishes throughout the world [5,6,7]. Their genomes contain two single-stranded RNAs and RNA2, and RNA1 encodes the RNA-dependent RNA polymerase (RdRp) while RNA2 encodes the coat protein (CP), respectively [8]. RNA1 is longer than RNA2. NNVs have been classified into four primary genotypes: striped jacked NNV, red-spotted grouper NNV (RGNNV), barfin flounder NNV, and tiger puffer NNV [5]. An effective method for preventing grouper virus disease is urgently required, and improving the immunity of the grouper is the most promising approach to the prevention and treatment of viral infections.

Tumor necrosis factor receptor (TNFR)-associated factors (TRAFs) are considered the central signal transducers of some signaling pathways and play important roles in some biological processes such as immune regulation, inflammatory response, and apoptosis [9,10]. Seven members of the TRAF family have been identified, designated TRAF1–7 [11]. Most TRAFs (except TRAF7) contain a TRAF domain in the C-terminal region and a C-terminal β-sandwich domain (TRAF-C or MATH domain) [12,13]. TRAFs also contain an N-terminal RING finger domain followed by multiple zinc finger motifs [12,14]. They determine the E3 ligase activity of the TRAFs and are crucial for the activation of downstream signaling cascades [15]. TRAF4 was first cloned from breast cancer-derived metastatic lymph nodes [16,17]. TRAF4 contains a nuclear localization signal (NLS), which is a unique member of the TRAF family [17,18]. TRAF4 has a RING domain and an E3 ubiquitin ligase domain, so it can mediate activation of the target proteins of TAK1 and AKT1 and the K63-linked ubiquitination [19,20]. TRAF4 interacts with the deubiquitinase USP10 and blocks the access of tumor protein P53 (TP53) to USP10, destabilizing TP53 [16]. Unlike other proteins of the TRAF family, TRAF4-deficient mice display impaired neural tube closures and tracheal ring disruptions [21]. TRAF4 can also interact weakly with the human p75 neurotrophin receptor (p75-NGFR), a member of the TNF-R present in the nervous system, and with a lymphotoxin-beta receptor (LTp-R) [22]. TRAF4 increases NF-κB activation through glucocorticoid-induced TNF-R (GITR). This effect is mediated by a TRAF-binding site located in the cytoplasmic domain of GITR and is inhibited by cytoplasmic protein A20 [23].

Compared with other teleost TRAF proteins, little is known of teleost TRAF4. To examine the roles of TRAF4 in the innate immunity of teleosts, the TRAF4 homologue of the orange-spotted grouper *(EcTRAF4)* was characterized in the present study. Next, the expression profiles of EcTRAF4 were analyzed in the tissues of healthy fish and in GS cells after viral infection. Finally, the effects of overexpressed EcTRAF4 on RGNNV proliferation were investigated.

## 2. Results

### 2.1. Identification and Sequence Analysis of EcTRAF4

**Figure 1:**
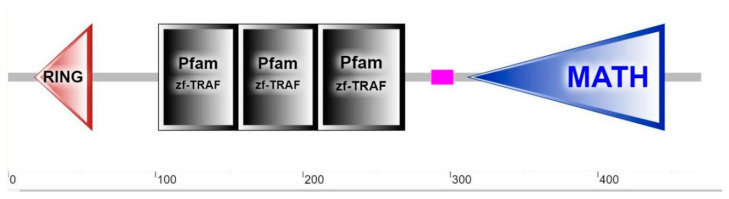
Putative conserved domains of EcTRAF4. One RING domain, three zinc finger domains, and one MATH domain were predicted by SMART (http://smart.embl-heidelberg.de/, accessed on 1 May 2021).

*EcTRAF*4 consisted of 1413 nucleotides encoding a 471-amino-acid protein, with a predicted molecular mass of 54.27 kDa. No signal peptide or transmembrane helices were detected in the deduced amino acid sequence of EcTRAF4. Like its mammalian counterparts, EcTRAF4 contained an N-terminal RING finger domain, three zinc finger domains, and a MATH domain (Figure 1).

**Figure 2:**
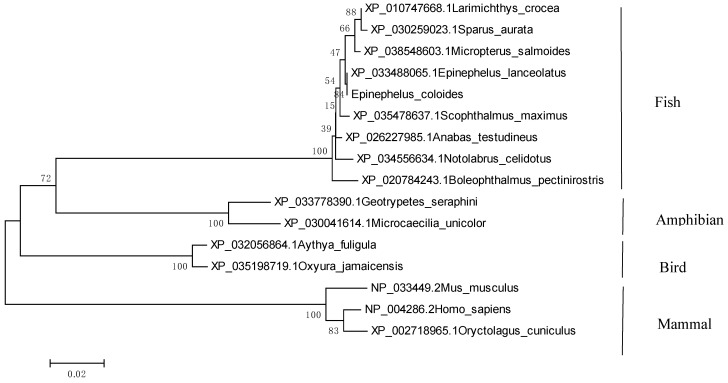
Phylogenetic analysis of the EcTRAF4 proteins. The GenBank accession number for each species is listed to the left of the species name. The phylogenetic tree was constructed using MEGA software 4.0. The relationships among the various components were analyzed by the neighbor-joining (NJ) method. Numbers on the branches indicate percent bootstrap confidence values from 1000 replicates.

EcTRAF4 shared 99.79% identity with TRAF4 of the giant grouper *E. lanceolatus*. A multiple sequence alignment was constructed with ClustalX1.83 software. On a neighbor-joining tree of TRAF4 proteins, EcTRAF4 clustered with the giant grouper TRAF4. The grouping of TRAF4 proteins was supported well by bootstrapping and was in accordance with the assumed evolutionary trends of the species represented (Figure 2).

### 2.2. Tissue Expression Analysis of EcTRAF4

**Figure 3:**
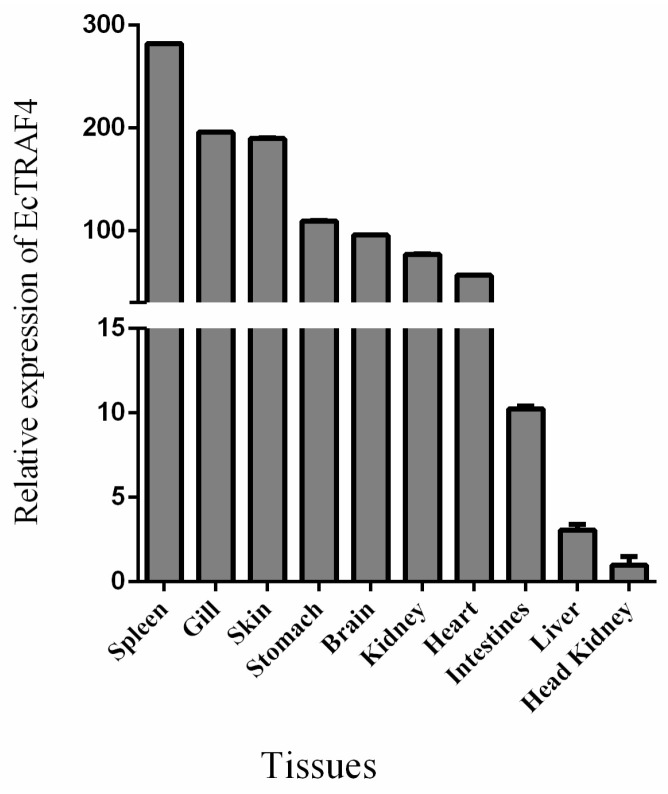
Tissue distribution of EcTRAF4 in healthy grouper. β-actin was used as the internal control. The expression of EcTRAF4 in the head kidney was set to 1.0. Data are expressed as the mean fold change (means ± S.E., *n* = 3) from the head kidney.

To investigate the tissue expression profile of *EcTRAF4* under normal physiological conditions, the total RNAs were extracted from 10 different tissues (kidney, heart, liver, spleen, intestine, stomach, brain, gill, head kidney, and skin) and analyzed with RT–qPCR. Figure 3 shows that *EcTRAF4* was expressed in all the tissues examined, but was predominantly expressed in the spleen, gill, skin, and stomach (Figure 3).

### 2.3. Intracellular Localization of EcTRAF4

**Figure 4:**
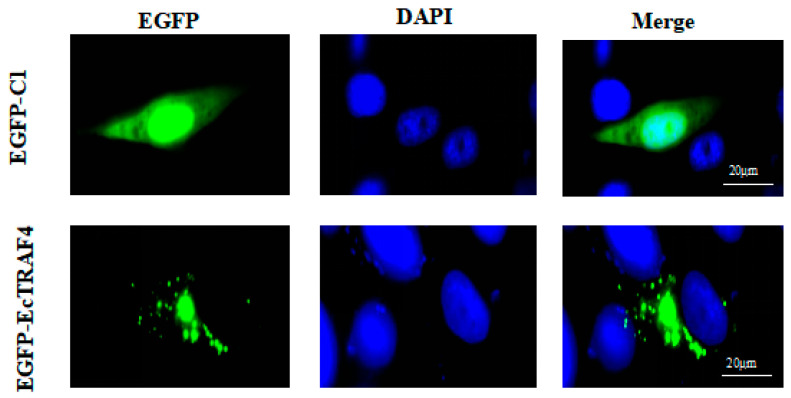
Subcellular localizations of EcTRAF4 in GS cells. GS cells were transfected with the plasmids of pEGFP–C1 and pEGFP–EcTRAF4 separately and then stained with DAPI. Samples were observed under fluorescence microscopy.

The intracellular localization of EcTRAF4 was determined by analyzing the expression of the enhanced green fluorescent protein (EGFP)–EcTRAF4 fusion protein. First, pEGFP–EcTRAF4 was constructed and used to transfect GS cells. The resulting fluorescent signal was observed with a fluorescence microscope. Fluorescence was equally distributed throughout the cytoplasm and nuclei in the pEGFP–C1-transfected cells, whereas in the pEGFP–EcTRAF4-transfected cells, green fluorescence was mainly observed in the cytoplasm (Figure 4).

### 2.4. EcTRAF4 Overexpression Promotes RGNNV Replication

**Figure 5:**
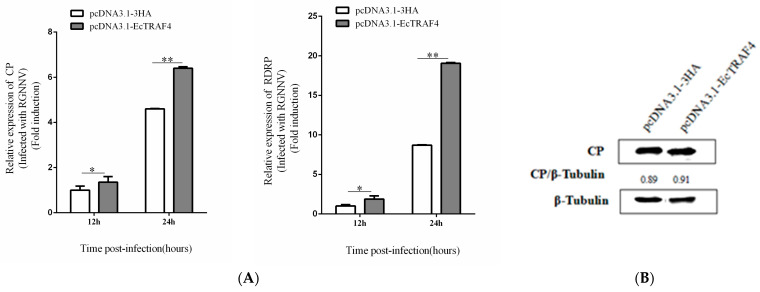
Effect of EcTRAF4 overexpression on RGNNV replication. (**A**) EcTRAF4 overexpression increased RGNNV gene transcription. Expression levels of CP and RdRp were measured using qRT-PCR (means ± S.E., *n* = 3). Statistical difference is with each pcDNA3.1-3HA-transfected cell. Asterisk denotes a significant difference (* *p* < 0.05 and ** *p* < 0.01). (**B**) Virus protein level after transfection with EcTRAF4. The level of RGNNV-CP was detected by western blot, and β-Tubulin was used as the internal control.

To confirm the effect of EcTRAF4 on RGNNV replication, *EcTRAF4*-transfected cells were infected with RGNNV, and viral replication was investigated. The cells were harvested after 12 or 24 h, and the transcription kinetics of the indicated RGNNV genes were measured with RT–qPCR. EcTRAF4 overexpression significantly increased the transcription of RGNNV genes (CP and RdRp) (Figure 5A). We also evaluated the effect of EcTRAF4 overexpression on viral protein synthesis. Consistent with the RT–qPCR results, the level of RGNNV CP protein was higher in the EcTRAF4-overexpressing cells than in the control cells (Figure 5B).

### 2.5. EcTRAF4 Knockdown Inhibits RGNNV Replication

**Figure 6:**
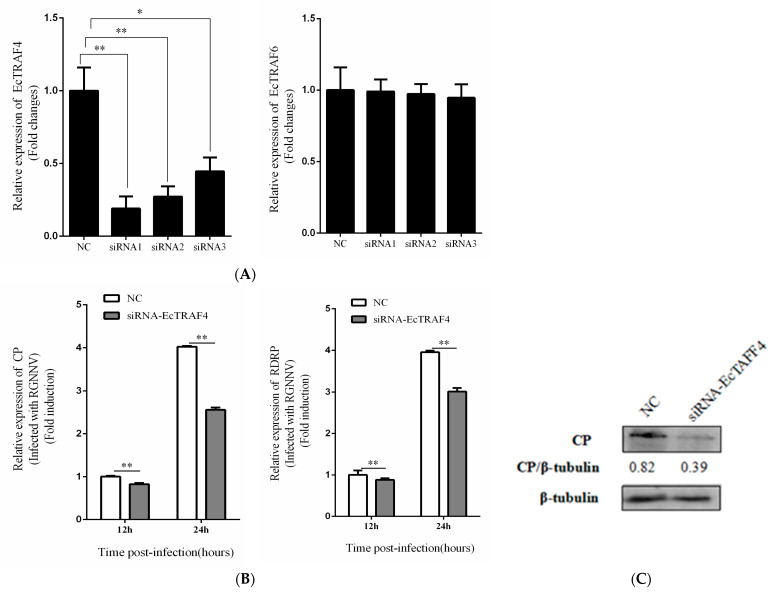
Effect of EcTRAF4 knockdown on RGNNV replication. (**A**) Three siRNA sequences were designed based on the sequence of EcTRAF4, the expression of EcTRAF4 was tested, and EcTRAF6 was used as the control (means ± S.E., *n* = 3). (**B**) Decreased RGNNV gene transcription, including CP and RdRp (means ± S.E., *n* = 3). Statistical difference is with each NC at a different time. Asterisk denotes a significant difference (* *p* < 0.05 and ** *p* < 0.01) (**C**) Virus protein level after transfection with siRNA1 of EcTRAF4. The level of RGNNV-CP was detected by western blot, and β-Tubulin was used as the internal control.

To determine the relationship between EcTRAF4 and RGNNV replication, we determined RGNNV replication in EcTRAF4-knockdown GS cells. Three siRNAs directed against EcTRAF4 mRNA were designed, and their knockdown efficiencies were determined. As shown in Figure 6A, siRNA1 had the highest knockdown efficiency (70%) in GS cells, and siRNAs had no efficiency to EcTRAF6. Therefore, we used siRNA1 to study the effects of silencing EcTRAF4 on RGNNV replication. An RT–qPCR analysis showed that the transcription levels of the RGNNV CP and RdRp genes decreased after EcTRAF4 knockdown (Figure 6B). We also evaluated the effects of EcTRAF4 knockdown on viral protein synthesis. Consistent with the RT–qPCR results, the RGNNV CP protein levels were lower in the EcTRAF4-knockdown cells than in the control cells (Figure 6C).

### 2.6. RGNNV CP Interacts with EcTRAF4

**Figure 7:**
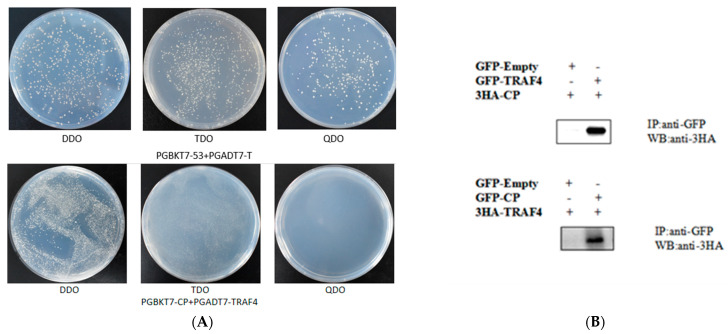
Interaction of CP with EcTRAF4. (**A**) For yeast two-hybrid analysis, pGBKT7-CP and pGADT7-EcTRAF4 plasmids were constructed for interaction verification. The two plasmids were co-transformed into S. cerevisiae strain Y2H Gold. The transformants were tested on a non-selective medium plate SD/-leu/-trp (DDO/X) to check whether the transformation was successful and the selective medium plate SD/-leu/-trp/-his/-ade/X-α-gal/AbA (QXA) to detect whether there was interaction between two proteins. (**B**) Co-immunoprecipitation resulted in GS cells showing that EcTRAF4 might interact with CP.

To further investigate the function of EcTRAF4, we examined the relationship between CP and EcTRAF4. First, a prey vector containing full-length *traf5* was constructed, and the EcTRAF4–CP interaction was verified with a yeast two-hybrid system. Yeast strain Y2H Gold was co-transformed with prey plasmid AD–EcTRAF4 and bait plasmid BD–CP or BD. The cells co-transformed with AD–EcTRAF4/BD-CP and BD-p53/AD-T grew on QDO/X/A plates, indicating an interaction between EcTRAF4 and CP (Figure 7A).

To confirm the EcTRAF4–CP interaction, coimmunoprecipitation (co-IP) experiments were performed by co-expressing EcTRAF4–GFP and CP–HA. After incubation with Anti-GFP M2 Affinity Gel, EcTRAF4–GFP successfully precipitated CP–HA (Figure 7B). These results, together with the yeast two-hybrid and co-IP results, demonstrated the interaction between EcTRAF4 and CP.

### 2.7. RGNNV or CP Promotes EcTRAF4 Expression

**Figure 8:**
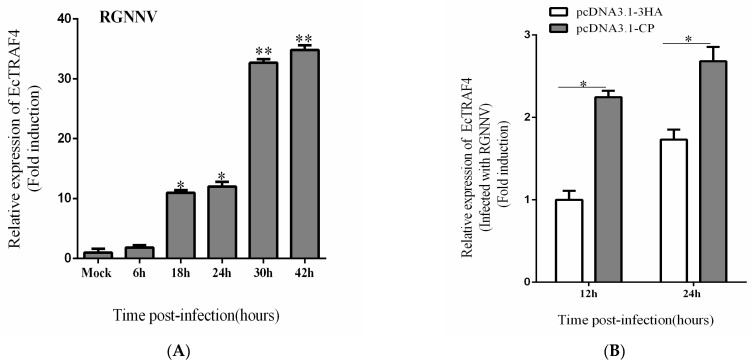
RGNNV and CP promoted EcTRAF4 expression. (**A**) Expression changes of EcTRAF4 in RGNNV-infected cells (means ± S.E., *n* = 3). β-actin was used as the internal control. Statistical difference is with mock. (**B**) EcTRAF4 mRNA in CP-expressing GS. GS were transfected with CP. TRAF4 mRNA expression was analyzed by RT–qPCR at 12 and 24 h. Asterisk denotes a significant difference (* *p* < 0.05 and ** *p* < 0.01).

After we confirmed the positive effect of EcTRAF4 on RGNNV replication, we investigated the effect of RGNNV infection on *EcTRAF4* expression. In response to RGNNV infection, the transcription of *EcTRAF4* in GS cells was higher than that in uninfected cells (Figure 8A). Because EcTRAF4 interacted with RGNNV CP, the effect of CP on *EcTRAF4* expression was investigated. As shown in Figure 8B, EcTRAF4 mRNA was increased in GS cells expressing CP, indicating that RGNNV or CP increased cellular *EcTRAF4* expression. Together with the fact that EcTRAF4 promoted RGNNV propagation, these data implied that RGNNV exploited EcTRAF4 via CP to improve its proliferation and that EcTRAF4 played an important role in RGNNV infection.

### 2.8. EcTRAF4 Is a Negative Regulator of Virus-Induced IFN Signaling

**Figure 9:**
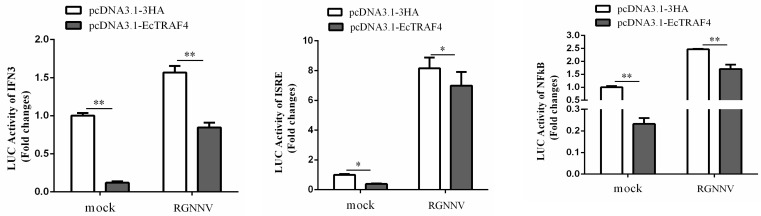
The relative luciferase activity of IFN, ISRE, and NF-κB promoter in EcTRAF4-overexpressing cells. GS cells were co-transfected with NF-κB-Luc/IFN-β-Luc/ISRE-Luc, pEGFP-C1, and pEGFP-EcTRAF4. Twenty-four hours after transfection, cells were infected with RGNNV or left uninfected for 20 h before luciferase assays were performed (means ± S.E., *n* = 3). Asterisk denotes a significant difference (* *p* < 0.05 and ** *p* < 0.01).

To investigate whether EcTRAF4 was involved in the regulation of virus-induced IFN signaling, GS cells were co-transfected with 200 ng of plasmid-encoding ISRE–Luc, IFN3–Luc, or NF-κB-Luc and 600 ng of pcDNA3.1-3×HA or plasmid-encoding HA–EcTRAF4. A total of 50 ng of pRL-SV40 Renilla luciferase vector (Promega, USA) was used as the internal control. The cells were harvested after 48 h to measure the luciferase activities with the Dual-Luciferase^®^ Reporter Assay System. As shown in Figure 9, the overexpression of EcTRAF4 inhibited the RGNNV-induced activation of IFN3, the ISRE, and NF-κB.

**Figure 10:**
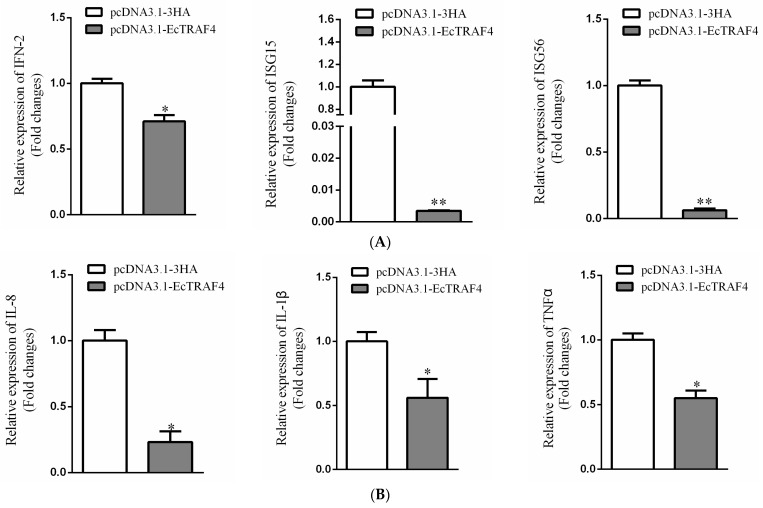
Relative expression levels of IFN-signaling molecules and proinflammatory cytokines in EcTRAF4-overexpressing cells. (**A**) Expression changes of IFN-signaling molecules (ISG15, ISG56 and IFN-2) in EcTRAF4-overexpressing cells (means ± S.E., *n* = 3). (**B**) Expression changes of inflammatory factors (IL-1β and TNFα) in EcTRAF4-overexpressing cells (means ± S.E., *n* = 3). β-actin was used as the internal control. The asterisk denotes a significant difference (* *p* < 0.05 and ** *p* < 0.01).

To investigate the mechanisms involved in the action of EcTRAF4 during viral infection in fish, we evaluated the roles of EcTRAF4 in the host IFN-mediated immune response and inflammatory response. At 24 h after transfection, the expression levels of IFN or inflammation-related genes (ISG15, ISG56, IFN-2, TNFα, IL-1β, and IL-8) were measured with RT–qPCR. The expression of all genes was significantly reduced in the EcTRAF4-overexpressing cells compared with their expression in the control vector-transfected cells (Figure 10A,B). Taken together, these data indicated that EcTRAF4 was a negative regulator of virus-induced IFN signaling.

## 3. Discussion

As an important family of signal transduction proteins, the TRAF proteins have been found in all animal species [24]. Although several TRAF family members in teleosts had been studied, and the results showed that they played important roles in the innate immune system [25,26,27,28], the function of TRAF4 in the orange-spotted grouper had not been established.

In this study, the *TRAF4* homologue in the orange-spotted grouper, *EcTRAF4*, was cloned. Similar to its mammalian counterparts, the EcTRAF4 protein includes one N-terminal RING finger domain, three zinc finger domains, and a MATH domain. In our previous study, TRAF6 from *E. tauvina* (Et-TRAF6) also includes one N-terminal RING domain (amino acids 78–116), two TRAF-type zinc fingers (amino acids 159–210 and 212–269), one coiled-coil region (amino acids 370–394), and one conserved C-terminal meprin and TRAF homology (MATH) domain (amino acids 401–526) [26]. The EcTRAF4 and EtTRAF6 proteins apparently share a similar motif composition, indicating that they may have potential functional similarities.

In a previous study, fish TRAF genes were shown to be widely expressed in the examined tissues [24]. Most TRAF genes were highly expressed in the gills. Gills mediate the contact between aquatic organisms and their external aquatic environments. Gills are also the first defensive barriers to pathogen invasions [29]. *TRAF* genes were also detected at higher levels in the liver and spleen. The liver and spleen are the important immune-related tissues in teleost fish [30]. To investigate the tissue expression profile of *EcTRAF4* under normal physiological conditions, 10 different tissues from healthy grouper were analyzed with RT–qPCR. EcTRAF4 was expressed in all the examined tissues and predominantly expressed in the spleen, gill, skin, and stomach, similar to most fish *TRAF* genes [24,26,28]. To investigate the involvement of TRAFs in the innate immune responses of teleost fish, the expression profile of EcTRAF4 in GS cells after challenges with RGNNV was examined. In response to RGNNV infection, the transcription of EcTRAF4 in GS cells was higher than in the uninfected cells. As the infection time increased, its expression level gradually increased.

EcTRAF4 could interact with RGNNV CP, and the expression of EcTRAF4 mRNA was increased in GS cells expressing CP. These results indicated that EcTRAF4 was involved in the immune response to the invasion of viral pathogens. In previous studies, TRAF5 was shown to interact with NS3 and promoted *classical swine fever virus* (CSFV) replication [27]. Similarly, TRAF5 also interacted with the Human Immunodeficiency Virus-1 (HIV-1) Nef protein and HIV-1 Nef-activated TRAF5 to promote HIV-1 replication in monocyte-derived macrophages [31]. To investigate the molecular mechanism underlying the host antiviral innate immune response, the effects of EcTRAF4 overexpression on RGNNV replication were studied. Overexpressed EcTRAF4 promoted RGNNV replication and reduced the transcription levels of genes encoding IFN-related cytokines (ISG15, ISG56, and IFN-2) and proinflammatory cytokines (IL1β, IL8, and TNFα). EcTRAF4 also inhibited the RGNNV-induced activation of IFN3 and ISRE. Therefore, EcTRAF4 negatively regulated the IFN-mediated immune response and the expression of proinflammatory cytokines to facilitate RGNNV proliferation.

The expression of TRAF4 has different distribution in different cells [32]. Some have reported that TRAF4 is a dynamic, tight junction-related shuttle protein [33], whereas other researchers have thought that different structural domains influence cellular localization [34]. In the present study, the localization of EcTRAF4 in GS cells was investigated with fluorescence microscopy to preliminarily determine whether TRAF4 remained in the cytoplasm as a signal transducer. Consistent with previous studies, our results showed that EcTRAF4 localized mainly in the cytoplasm of GS cells, and all the cells showed a similar appearance under a 10× microscope.

It was reported that TRAF family members in mammals were involved in the innate immune and inflammatory responses to mediate the MAPK, IRF, and NF-κB pathways [15,24,26,28]. Studies in mammals have reported that TRAF4 promotes NF-κB activation by the GITR protein but inhibits the NF-κB activation induced by LPS or the binding of NOD2 [35]. In contrast, TRAF4 activates NF-κB in HEK-293 cells in the amphioxus [36]. In the present study, we found that EcTRAF4 inhibited the RGNNV-induced activation of NF-κB.

In conclusion, the complete ORF of *EcTRAF4* was cloned. The sequence, critical functional domains, and phylogeny were conducted with bioinformatic methods. We also analyzed the expression of EcTRAF4 in the tissues of grouper and in GS cells after viral infection. EcTRAF4 was also localized intracellularly, and the effect of EcTRAF4 overexpression on RGNNV proliferation was investigated. EcTRAF4 was then overexpressed in GS cells to determine whether it activated IFN, ISRE, and NF-κB. The results of this study provide valuable information, allowing a comprehensive understanding of the evolution of fish TRAF4 and its functions in the innate immune system.

## 4. Materials and Methods

### 4.1. Fish, Cell Lines, and Virus

Healthy orange-spotted grouper (weighing 30–40 g) were purchased from Maoming Marine Fish Farm, Guangdong Province, China. The fish were maintained in the laboratory in a recirculating seawater system at 24–28 °C and fed twice daily for 2 weeks [37]. Tissue samples from six fish including kidney, heart, liver, spleen, intestine, stomach, brain, gill, head kidney, and skin samples were excised, immediately frozen in liquid nitrogen, and stored at −80 °C [37].

Grouper spleen (GS) cells were propagated with the recommended method in Leibovitz’s L15 culture medium, containing 10% fetal calf serum, at 28 °C [38]. Red-spotted grouper nervous necrosis virus (RGNNV) was propagated as described previously [39,40]. The viral titer was 10^5^ TCID_50_/mL.

The GS cells were challenged with RGNNV (multiplicity of infection (MOI) = 2) for 6, 18, 24, 30, or 42 h. The RNA was then extracted from the cells to determine the expression of *EcTRAF4* with reverse transcription (RT)–quantitative real-time PCR (qPCR). Unchallenged GS cells were used as the control.

### 4.2. RNA Isolation, cDNA Synthesis, and RT–qPCR

According to the manufacturers’ instructions, RNA extraction and cDNA synthesis were performed with the SV Total RNA Isolation System (Promega, Madison, USA) and ReverTra Ace qPCR RT Kit (Toyobo, Osaka, Japan), respectively. RT–qPCR was performed with SYBR^®^ Green Realtime PCR Master Mix (Toyobo) in an Applied Biosystems QuantStudio 5 Real Time PCR System (Thermo Fisher, MA, USA), as described previously [37,40]. Briefly, each assay was performed in triplicate with the following cycling conditions: 95 °C for 1 min for activation, followed by 40 cycles of 95 °C for 15 s, 60 °C for 15 s, and 72 °C for 45 s. The expression levels of the target genes were normalized to that of β-actin and calculated with the 2^−^^△△CT^ method. The data are presented as means ± standard deviations (SD). The primer sequences used for RT–qPCR are listed in Table 1.

### 4.3. Cloning of EcTRAF4 and Sequence Analysis

The ORF of *EcTRAF4* was amplified with PCR, using primers that were designed based on the sequence of *Epinephelus coioides* TRAF4 (KR005609.1). The primers are listed in Table 2. The sequence of *EcTRAF4* was analyzed with the BLAST program (http://www.ncbi.nlm.nih.gov/blast, accessed on 1 May 2021). An amino acid sequence alignment was constructed with ClustalX1.83 software and was edited with the GeneDoc program. The neighbor-joining (NJ) method implemented in MEGA 4.0 was used for a phylogenetic analysis. The robustness of the phylogenetic tree bifurcations was estimated with a bootstrap analysis, and the bootstrap percentages were calculated from 1000 replicates.

### 4.4. Plasmid Construction

The ORF of *EcTRAF4* was subcloned into the pcDNA3.1-3HA or pEGFP-C1 vector (Invitrogen) to generate the recombinant plasmids pcDNA3.1–EcTRAF4 and pEGFP–EcTRAF4, respectively. The ORF of the coat protein (CP) of RGNNV was subcloned into the pcDNA3.1-3HA vector (Invitrogen) to generate the recombinant plasmid pcDNA3.1–CP. The recombinant plasmids were confirmed with DNA sequencing. The primers used to amplify these genes are listed in Table 2.

### 4.5. Cell Transfection

Cells were transfected using Lipofectamine 2000 (Invitrogen), as described previously [36,39]. Briefly, GS cells (5 × 10^5^) were seeded in 24-well plates or 6-well plates, grown to 70–80% confluence, and then incubated with a mixture of Lipofectamine 2000 and plasmid for 6 h. The mixture then was replaced with fresh normal medium.

### 4.6. Cellular Localization Analysis

GS cells (5 × 10^5^) were seeded onto coverslips (10 mm × 10 mm) in a six-well plate. After the cells were allowed to adhere for 24 h, they were transfected with the pEGFP–EcTRAF4 or pEGFP–C1 plasmid. At 24 h post-transfection, the cells were washed with phosphate-buffered saline, fixed with 4% paraformaldehyde for 1 h, and then stained with 6-diamidino-2-pheny-lindole (DAPI) for 10 min. They were then mounted with 50% glycerol and observed with fluorescence microscopy (Leica, Germany).

### 4.7. Viral Infection Assays and Sample Collection

To evaluate the effects of EcTRAF4 on viral replication, GS cells overexpressing pcDNA3.1 or pcDNA3.1-EcTRAF4 were infected with RGNNV (MOI = 2). Next, the cells were harvested and analyzed by RT–qPCR and western blotting [37].

### 4.8. Small Interfering RNA (siRNA)-Mediated EcTRAF4 Knockdown

To knockdown the expression of EcTRAF4 in GS cells, three siRNAs, targeting different parts of the EcTRAF4 mRNA molecule, were commercially synthesized by Invitrogen. The sequences of siRNAs were as follows: siRNA1 (sense: 5′-GCUAACCAUGUGAAGGACATT-3′; antisense: 5′-UUGUGUCGAAGACGAACUCTT-3′), siRNA2 (sense: 5′-UCUCCUACAAGGUGACUUUTT-3′; antisense: 5′-AAAGUCACCUUGUAGGAGATT-3′), and siRNA3 (sense: 5′-GCUAACCAUGUGAAGGACATT-3′: antisense: 5′-UGUCCUUCACAUGGUUAGCTT-3′). One of these three siRNAs or the same amount of the negative control siRNA were transfected into GS cells, and then infected with RGNNV or left untreated. At the end of the incubation period, the total RNA was extracted from the cells and analyzed with RT–qPCR and western blotting [37].

### 4.9. Coimmunoprecipitation Assays and Western Blotting

GS cells in cell culture dishes (10 cm × 10 cm) were transfected with 16 μg of plasmid DNA (8 μg of each expression vector) for 48 h. The transfected GS cells were lysed in radioimmunoprotein assay buffer (containing 100 mM NaCl, 0.5% NP-40, 1 mM EDTA, 20 mM Tris; pH 8.0). The Dynabeads™ Protein G Immunoprecipitation Kit (Invitrogen) was used to process the collected cell samples. The proteins were separated with 12% SDS-PAGE and transferred onto Immobilon-P polyvinylidene difluoride membranes (Millipore, Temecula, CA, USA). The blots were incubated with an anti-green fluorescent protein (GFP, diluted 1:1000), anti-3 × hemagglutinin (3HA, diluted 1:1000), or anti-RGNNV CP primary antibody (diluted 1:1000) and then with a horseradish peroxidase (HRP)-conjugated anti-rabbit IgG antibody (diluted 1:5000) or an HRP-conjugated anti-mouse IgG antibody (diluted 1:5000). The immunoreactive proteins were observed with the Enhanced HRP-DAB Chromogenic Substrate Kit (Tiangen, Beijing, China) [40].

### 4.10. Yeast Two-Hybrid Analysis

For the yeast two-hybrid analysis, the corresponding genes were cloned separately into the pGBKT7 and pGADT7 vectors, and the pGBKT7-BD and pGADT7–EcTRAF4 plasmids were constructed for the verification of self-activation. pGBKT7-CP was also constructed for the verification of interaction. All constructed plasmids were confirmed with DNA sequencing. *Saccharomyces cerevisiae* strain Y2H Gold was co-transformed with the two plasmids. The transformants were tested on SD/-leu/-trp and SD/-leu/-trp/-his/-ade/X-α-gal/AbA media.

### 4.11. RT–qPCR Analysis of Relative Expression Levels of host and Viral Genes

To examine the transcriptional expression of host and viral genes, RT–qPCR was performed, as described above [37,40]. The primers for the amplification of the host IFN-signaling molecules (ISG15, ISG56, and IFN-2), host proinflammatory factors (IL-1β, IL-8, and TNFα), and viral genes (CP and RdRp) were reported previously [40] and are listed in Table 2. The expression levels of the target genes were calculated with the 2^−^^ΔΔCT^ method, and gene-encoding β-actin was used as the reference gene. Samples transfected with the empty vector were used as the calibrator group against which to normalize gene expression. The data are represented as means ± standard errors of the means (SEM).

### 4.12. Dual-Luciferase Reporter Assays

To examine the effects of EcTRAF4 on the activity of IFN and the nuclear factor-κB (NF-κB) promoter, luciferase plasmids, including those encoding zebrafish IFN3–Luc, human IFN-stimulated response element (ISRE)–Luc, and NF-κB–Luc (Promega, Madison, WI, USA), were used [37,40]. In brief, GS cells were co-transfected with 200 ng of plasmid-encoding ISRE–Luc, IFN3–Luc, or NF-κB-Luc and 600 ng of pcDNA3.1-3 × HA or plasmid-encoding HA–EcTRAF4. A total of 50 ng of pRL-SV40 Renilla luciferase vector (Promega, Madison, WI, USA) was used as the internal control. The cells were harvested after 48 h to measure luciferase activities with the Dual-Luciferase^®^ Reporter Assay System (Promega, Madison, WI, USA), according to the manufacturer’s instructions.

### 4.13. Statistical Analyses

All statistics were calculated using SPSS version 20. Differences between control and treatment groups were assessed by one-way ANOVA. Differences were considered significant at *p* < 0.05 and highly significant at *p* < 0.01.

### 4.14. Ethics Statement

All experiments involving animals were approved by the Animal Care and Use Committee of the College of Marine Sciences, South China Agricultural University, and all efforts were made to minimize animal suffering. The ID number is HYXY20210328 (SCAU).

## Figures and Tables

**Table 1:** Primers used for host and viral gene expression analysis.

Name	Sequence (5′–3′)
RGNNV-CP-RT-F	CAACTGACAACGATCACACCTTC
RGNNV-CP-RT-R	CAATCGAACACTCCAGCGACA
RGNNV-RdRp-RT-F	GTGTCCGGAGAGGTTAAGGATG
RGNNV-RdRp-RT-R	CTTGAATTGATCAACGGTGAACA
EcTRAF4-RT-F	TTCAGCCCACCCTTCTACACTCA
EcTRAF4-RT-R	CCACTCCAGCAGGTTGTCGTACTCT
β-actin-RT-F β-actin-RT-R	TACGAGCTGCCTGACGGACA GGCTGTGATCTCCTTCTGCA
EcISG15-RT-F	CCTATGACATCAAAGCTGACGAGAC
EcISG15-RT-R	GTGCTGTTGGCAGTGACGTTGTAGT
EcISG56-RT-F	CTGTTGTTACGCACGGAGGAT
EcISG56-RT-R	CCTGCGTGGGTTCATTCAGT
EcIFN2-RT-S	TACAGCCAGGCGTCCAAAGCATC
EcIFN2-RT-R	CAGTACAGGAGCGAAGGCCGACA
EcIL-1β-RT-F	AACCTCATCATCGCCACACA
EcIL-1β-RT-R	AGTTGCCTCACAACCGAACAC
EcIL8-RT-F	GCCGTCAGTGAAGGGAGTCTAG
EcIL8-RT-R	ATCGCAGTGGGAGTTTGCA
EcTNFα-RT-F	GTGTCCTGCTGTTTGCTTGGTA
EcTNFα-RT-R	CAGTGTCCGACTTGATTAGTGCTT
EcTRAF6-RT-F	CCCTATCTGCCTTATGGCTTTGA
EcTRAF6-RT-R	ACAGCGGACAGTTAGCGAGAGTAT

**Table 2:** Primers used for EcTRAF4 cloning and plasmid construction.

Name	Sequence (5′–3′)	Usage
F1	ATGCCCGGGTTTGATTACAAGTTTC	EcTRAF4 cloning
R1	TTAAGCCATGATCTTCTGGGGAATC	
C1-EcTRAF4-F C1-EcTRAF4-R	GCCTCGAGCTATGCCCGGGTTTGATTAC GCGGATCCTTAAGCCATGATCTTCTGG	pEGFP-C1 cloning
HA-EcTRAF4-F	GCGGATCCTATGCCCGGGTTTGATTAC	pcDNA3.1-3HA cloning
HA-EcTRAF4-R	GCCTCGAGTTAAGCCATGATCTTCTGG	
HA-CP-F	GCAAGCTTATGGTACGCAAAGGTGAGAAGAAAT	pcDNA3.1-3HA cloning
HA-CP-R	GCGAATTCGTTTTCCGAGTCAACCCTGGTGCAG	

## Data Availability

Data supporting reported results can be provided by J.W. Wei.
